# SBMAC: Smart Blocking MAC Mechanism for Variable UW-ASN (Underwater Acoustic Sensor Network) Environment

**DOI:** 10.3390/s100100501

**Published:** 2010-01-12

**Authors:** Soo-Young Shin, Jung-Il Namgung, Soo-Hyun Park

**Affiliations:** The Graduate School of Business IT, Kookmin University, 861-1 Jeongneung-Dong Sungbuk-Gu, Seoul 136-702, Korea; E-Mails: sy-shin@kookmin.ac.kr (S.-Y.S.); greenji@naver.com (J.-I.N.G.)

**Keywords:** Underwater-Acoustic Sensor Network (UW-ASN), Medium Access Control (MAC), Smart Blocking MAC (SBMAC)

## Abstract

In this paper, several MAC scheduling methods applicable to an underwater environment are proposed. Besides, a new marine communication system model was proposed to improve the reliability of the proposed SBMAC method. The scheme minimizes transmission of control frames except for data transmission and various transmission methods and ACK methods can be used together. Simulation models are set indices and analysis of the underwater environment is established to conduct reliable simulations. Consequently, the performance improvement of the proposed method is verified with respect to delay time, data transmission rate, memory utilization, energy efficiency, *etc*.

## 1. Introduction

Unlike terrestrial wireless sensor networks that mainly rely on radio waves for communication, underwater sensor networks utilize acoustic waves, which present a much harsher environment for both the physical and the data-link layers. Acoustic waves appear to be a good choice for underwater communications because of their low loss when compared to radio waves. However, one major disadvantage is that acoustic waves travel at approximately 1,500 m/s, which is five orders of magnitude slower than radio waves. Moreover, the underwater acoustic channel’s bandwidth is very limited, typically in the order of several kilohertz. These undesirable characteristics are most significant at the data-link layer, because of the long propagation delay and packet transmission time [1].

Underwater Sensor Networks are typically distributed in the natural environment and the nodes communicate using acoustic waves over a wireless medium. Such networks are characterized by long and variable propagation delays, intermittent connectivity, limited bandwidth and low bit rates [2].

The terrestrial sensor network MAC protocols have been modified in order to adapt them to the differences of acoustic transmission in underwater environment. In [1], the authors studied the performance of ALOHA based protocols in underwater networks, and proposed two enhanced schemes, namely ALOHA with collision avoidance (ALOHA-CA), and ALOHA with avoidance notification (ALOHA-AN), which are capable of using the long propagation delays to their advantage. In [3], based on a channel access discipline called floor acquisition multiple access (FAMA) which combines both carrier sensing (CS) and dialogue between the source and receiver prior to data transmission, the Slotted FAMA was proposed to reduce excessive waste of control packets. On the other hand, many new scheduling and time synchronization methods have been proposed to solve the problem of large propagation delays and transmission variance, which is proportional to distance. In [4], the authors focused on designing an energy-efficient MAC protocol for a short range, acoustic sensor networks called “Tone Lohi.” Lohi provides an energy conserving, throughput efficient, fair, and stable medium access for acoustic networks. In [5], the authors presented a distributed, scalable, energy-efficient MAC protocol that works despite long, unknown propagation delays of the underwater acoustic medium. This protocol can be used for delay-tolerant applications such as underwater ecological sensor networks between energy-limited nodes. Most of the proposed protocols want to solve synchronization problems and minimize the length of hand-shake procedure for non-synchronized *ad hoc* UWASN.

In a field of MAC for underwater sensor networks, Channel access control and Multiple access control suitable to underwater condition are effective methods for processing limited bandwidth in underwater condition and significant transmission delay. The goal looks similar to that of above ground sensor networks but the differences in their required levels are very large and their effects on the efficiency of devices are also different. A media access mechanism which is adaptive to underwater conditions must be proposed. Although the CDMA method, which has been mentioned as an alternative candidate, is capable of communication with many nodes and is insensitive to delay time, its complicated implementation and the policy tuning of original technologies should be taken into consideration [2]. It is thought that Cluster TDMA is a good method since the disadvantages of TDMA are minimized by using TDMA only inside a unit cluster [1]. Besides, the proposition of error model which is based on the analysis of traffic environment in underwater condition and calculation of proper size of packets by defining frame structure which is suitable to underwater condition are important research fields in this area.

In this paper, a new MAC technique which can operate adaptively in various underwater environments was proposed. The biggest advantage of the proposed technique is that an optimized transmission environment can be maintained continuously by determining transmission and re-transmission policies based on environmental variables. The concept of the proposed technique is explained in Chapter 2. Chapter 3 presents its mathematical model and a more detailed description of the technique. Simulation results comparing the proposed technique and conventional techniques are presented in Chapter 4 and final conclusions in Chapter 5.

## 2. Smart Blocking MAC

### 2.1. MAC System Model

In this paper, we selected clustered TDMA, which is applicable to a network with non-distant transmission range, as a transmission method. In this research, the TDMA method is a standard of multiple accesses and reservation is used as time slot allocation technique. MAC Scheduling is conducted dominantly by coordinator. A top priority of the research is given to the efficient use of MAC scheduling with respect to time and bandwidth. Once Master (coordinator) and Slaves (sensor nodes) are scattered in the ocean, all nodes maintain a power-on condition standby state. Then, Master broadcasts an Init_Start message, which includes sync, request duration and time stamp, to all nodes. Each sensor node calculates the time slot with respect to sync, includes MAC address and transmission start time into the time stamp, and transmits an Init_Response message three times during the RQD (Request Duration) time by way of slotted ALOHA - random access method. At this moment, the transmission trying time per unit RQD time is minimized so as to minimize the error rate of data transmission and reception for network configurations. After exceeding the first RQD, Master transmits an Init_Start message which includes the first MAC address, NodeID mapping list, the second RQD, sync and time stamp. At this time, sensor nodes who do not join the network can be additional members of the network by three transmissions times. After the second RQD, the Master broadcasts a final Init_Response message containing DGID (Distance Group ID), which is calculated with the Master-node distance which is measured using the MAC address, NodeID mapping list and propagation delay of the time stamps.

The initialization procedure of an underwater sensor network can be repeated with a long period depending on the system characteristics. Since the initialization procedure is conducted when new network members are added to or removed from the network or if there are network failures caused by a move or fault of the network, it supports modification, replacement or reconfiguration of new network members. A network reset is delivered to each member by using the NAV setting of the control field in a Beacon message [6].

A technique of dynamic cluster configuration is also possible with the assumption of an *Ad hoc* environment. However, continuous monitoring of dynamic changes of the network and frequent re-configuration procedure should be supported and consequently adequate processing of Initialization or Reconfiguration procedures will be needed and the network management workload will also be increased. This paper is not focused on network reconfiguration, mobility and flexibility but rather on reliable link-by-link data transmission capability. At this point of time, issues of 2D, 3D and mobility will be addressed in the next stage of our research.

### 2.2. Smart Blocking Model

The proposed SBMAC method is described briefly in this section. In underwater conditions, the most efficient transmission method is to minimize the number of data transmissions because, in underwater conditions, a long enough guard time, which is dependent on a distance, is required to guarantee a stable transmission time which results from the difficulty of synchronization between sender and receiver and large variation of transmission time delay and delay bandwidth. In a situation where all nodes have to be synchronized to start transmission or receipt of data, there is no choice but to determine the guard time on the basis of the most distant node. The distance between Master and the corresponding node can be measured by checking propagation delay times during the network initialization procedure and a guard time band, which is required for synchronization, can be set at this time.

**Figure 1.**
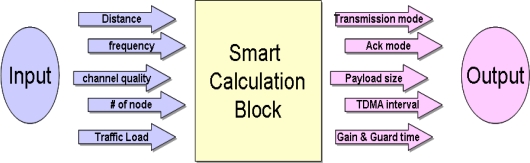
Smart Calculation Block diagram.

The principle subject of this paper is to propose an adaptive method of Smart Blocking Data and ACK transmission by SCB in underwater conditions. Smart Calculation Block, which is included in the Master, plays a leading role in determining the policies during network initialization and data transmission. The policies which SCB determines include the decision of TDMA transmission period, data transmission policy (*i.e.*, normal or blocked data), ACK policy (*i.e.*, No-ACK or SMA (Selective-Multiple-ACK) or RWA (Reduced-Whole-ACK) or MBA (Multiple-Block-ACK) or RBA (Reduced-Block-ACK)) and *etc*. Figure 1 shows a simplified diagram of these procedures.

### 2.3. Adaptive Transmission Model

The Master broadcasts the Beacon, which includes Transmission mode, Ack mode, TDMA interval information, Gain and Guard time, then all nodes who received the Beacon begin data transmission according to their own TDMA schedules. In the case of data transmission, both Normal-ACK and No-ACK transmit the same data but with a different ‘Smart Block type’. When the next Beacon is transmitted, whether or not there are transmission errors is notified by sending No-ACK against No-ACK required data, and by sending SMA (Selective Multiple ACK) against normal data. Transmission gain denotes the phenomenon that a margin for time caused by relatively long propagation delay makes Slave #2 to be able to transmit data transmission even though Slave #1 has not finished its transmission [7].

If scheduling is carefully conducted based on the time differences between each data transmission, the sharable time without any collision between channels can be found and used. This is a feasible concept when the distance between nodes are long enough and the length of transmitting data can be anticipated.

**Figure 3.**
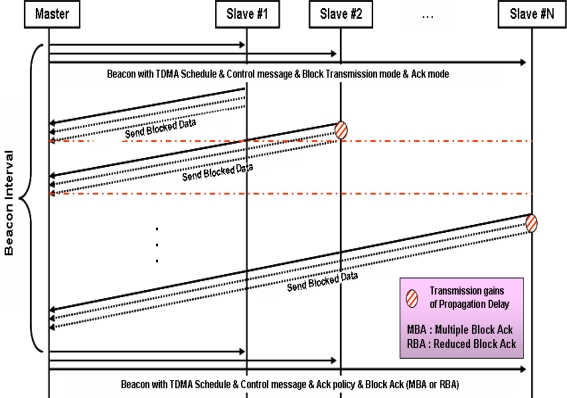
Blocked data transmission.

Figure 3 shows the procedure for blocked data transmission. Firstly, Master broadcast Beacon with TDMA info, control message and blocked or No-ACK policy. All nodes who received the Beacon transmit blocked data according to their TDMA schedule. When they transmit data, blocked data according to several MSDU policies are transmitted. In case of No-ACK, however, the setting is different depending on whether ‘Smart Block type’ is No-ACK or blocked ACK Then, when the next Beacon frame is transmitted, messages notifying whether or not there are transmission error is to be transmitted. As for the detection and the restoration of errors in underwater sensor systems, there are three methods : No-ACK, Normal-ACK and Smart-Block-ACK. No-ACK method can be used in case that the error rate of transmission route increases steeply or the characteristic of data is sensitive to the limit of time, such as the cases of audio data, continuous still images or the case that the need of data transmission exceeds the acceptable bandwidth of the whole networks resulted from a large amount of nodes in the networks. Normal-ACK method is used in case that the number of nodes is not so large and the amount of data to transmit is not large. In this case, Normal-ACK method can be used with lower power mode to maximize the network life time. Lastly, Smart-Block-ACK method can be used in case that there are large network load and efficient data transmission is also required continuously. And it is used to minimize transmitting control packets as well. The policy determination is to be made based on the overall analysis of network conditions.

## 3. SBMAC Mechanism

The starting point of the Smart Blocking MAC is to identify underwater conditions in a smart manner, calculate the Guard band, channel quality and bandwidth, and then determine an underwater communication policy and important parameters. The transmission policy determined by Smart Calculation can be classified into the Normal Data transmission method and the Blocked Data transmission method. In the case of Normal Data transmission, the receiver can use Selective Multiple Ack or No Ack method. In case of Blocked Data transmission, the receiver can use Multiple Block Ack or No Ack method. Although TDMA interval, Gain time, Guard time and Beacon interval are important concepts, they are not the point of this research now and still remain to be addressed in future work.

### 3.1. SBMAC System

**Figure 4.**
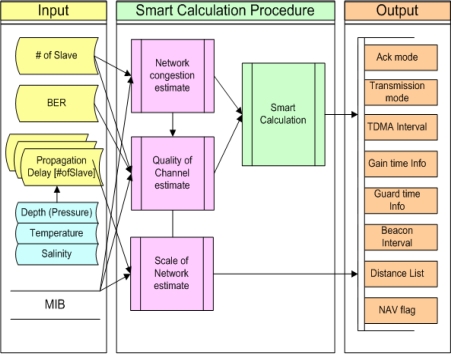
Smart Calculation Procedure.

In this section, the procedure for Smart Calculation is described in detail. By going through a Smart Calculation Block, important data which are related to policies of transmission and error restoration, TDMA, Congestion Control and Scheduling, are to be calculated based on many input variables. Figure 4 shows conceptual diagrams of the overall flow of Smart Calculation procedures.

Firstly, transmission delay time considering the number of Slaves managed by a Master, channel error rate, water depth, water temperature and salinity is measured and input. MIB information, which is a set of variables in MAC, is called out of necessity. Then, in the Smart Calculation Procedure, the Network congestion estimation process, which measures and samples the degree of congestion, and Quality of Channel estimation process, which measures channel quality based on transmission error rates, Scale of Network estimation process, which conducts Distance Grouping by measuring network scale and calculates TDMA intervals, standard value of Gain time and Guard time, and other processes are performed. Lastly, the Smart Calculation process is conducted for determination of transmission and error restoration policies.

As a result of computations, information of Ack mode, Transmission mode, TDMA interval, Gain time information, Guard time information, Beacon interval, Distance list and NAV flag will be generated. That is, Ack Transmission policy, calculated surplus time caused by various intervals and transmission, and other transmission indices will be obtained. It will be setup by calculating threshold value after identifying error rate and congestion rate during network initiation procedure, or the default system configuration value can also be used. MIB is a database storing variable values and, by changing these values, methods are provided to manage network managers’ requests dynamically.

The initialization procedure of an underwater sensor network can be repeated with a long period depending on the system characteristics. Since the initialization procedure is conducted when new network members are added to or removed from the network or if there are network failures caused by a move or fault of the network, modification, replacement or reconfiguration of new network members is supported. Network reset is delivered to each member by using the NAV setting of the control field in the Beacon message.

When the network is initialized, data transmission is conducted by means of MAC scheduling which is based on TDMA reservation. Firstly, the Master broadcasts a Beacon message with a BI (Beacon Interval) to transmit TDMA and other control information. Then Slaves conduct SBM (Smart Blocking MAC) Mechanism by adopting a data framing method and the application of the ACK-policy. In the next section, more detailed explanations on each calculation block are presented.

### 3.2. Network Congestion Estimation Process

**Figure 5.**
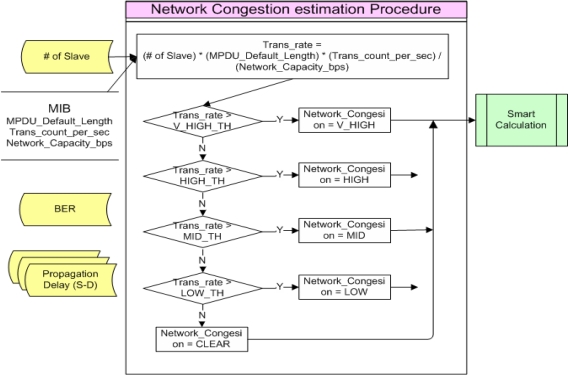
Network Congestion estimation Procedure.

During Network Congestion estimation process, the amount of generated Traffic versus bandwidth is estimated to calculate the degree of network congestion. By comparing the calculated results with channel bandwidth, the degree of network congestion of present channel load to be used for determination of transmission policy is identified. Figure 5 shows the procedure of Network Congestion estimation.

In the Network Congestion estimation process, the number of Slaves, MPDU_Defaut_Length, Trans_count_per_sec and Network_Capacity_bps which were stored in MIB are referred to calculate a Trans_rate. The calculated Trans_rate is compared with Threshold of V_HIGH_TH ∼ LOW_TH to calculate the degree of Network congestion. At this time, the degree of network congestion is divided into five levels, which are V_HIGH, HIGH, MID, LOW and CLEAR. They are used as comparison indices for Smart Calculation.

Each Trans_rate Threshold value (V_HIGH_TH ∼ LOW_TH), which are the basis for comparison, can be changed by a system administrator or system configuration values to make it be a more adaptive or robust system.

### 3.3. Quality of Channel Estimation Process

**Figure 6.**
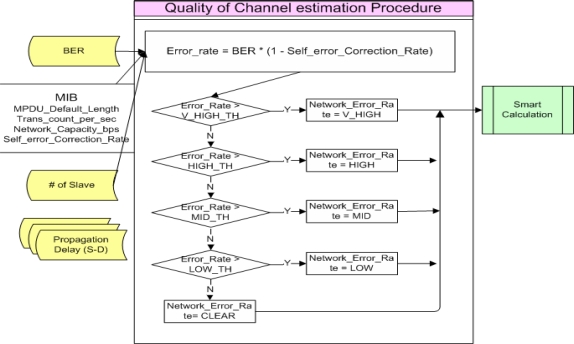
Quality of Channel estimation Procedure.

During the Quality of Channel estimation process, the error rate of each transmission channel is estimated to calculate the quality of the channel. At this time, BER is the stored transmission error rate during a certain constant duration time and the final estimated Error rate is obtained by excluding the probability of error restoration, such as CRC, *etc.* This value is compared with certain criteria to be used for determination of transmission policy. Figure 6 shows the Quality of Channel estimation process procedure.

In the Quality of Channel estimation process, the Error_Rate is calculated based on the number of Slaves, MPDU_Default_Length stored in MIB, Trans_count_per_sec, Network_Capacity_bps and Self_error_Correction_Rate. The calculated Error_Rate is then compared with the Threshold of V_HIGH_TH ∼ LOW_TH to calculate a Network_Error_Rate. At this time, the network transmission error rates are divided into V_HIGH, HIGH, MID, LOW and CLEAR. This value becomes a comparison index for Smart Calculation.

Each Error_Rate Threshold value (V_HIGH_TH ∼ LOW_TH), which are the basis for comparison, can be changed by a system administrator or system configuration values to make it be more adaptive or robust system.

### 3.4. Scale of Network Estimation Process

**Figure 7.**
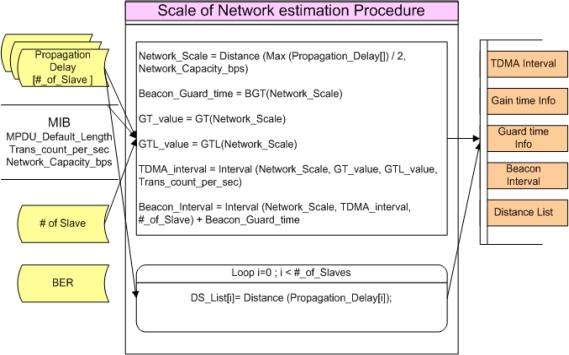
Scale of Network estimation Procedure.

During the Scale of network estimation process, Network Scale or Transmission radius of the network is measured and each slave’s relative distance from the Master is identified and a Distance Group is configured. Besides, Various Intervals for usage in various Scheduling is calculated. Figure 7 shows the Scale of network estimation process procedure.

In the Scale of Network estimation process, Network_Scale, Beacon_Guard_time, GT_value, GTL_value, TDMA_interval and Beacon_Interval are calculated based on the number of Slaves and MPDU_Default_Length, Trans_count_per_sec, Network_Capacity_bps which are stored in MIB. Besides, DS_List[] is generated according to Propagation_Delay which is generated between Slaves. Data, which is transmitted with Initial response and Beacon information, is then calculated.

### 3.5. Smart Calculation Procedure

**Figure 8.**
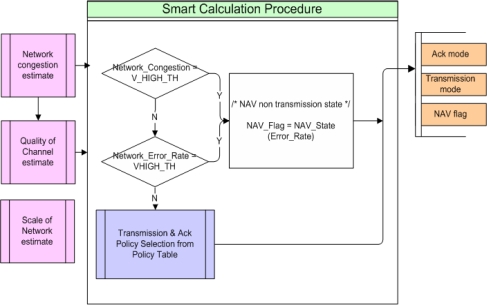
Smart Calculation Procedure.

**Figure 9.**
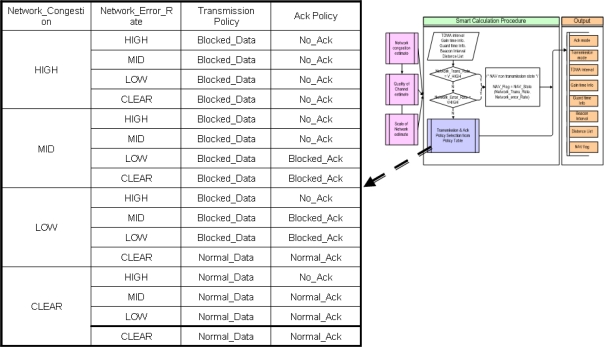
An example of Policy Table.

Figure 8 is a conceptual diagram of the Smart Calculation Procedure. In the Smart Calculation Procedure, NAV policy, Transmission policy and error restoration policy are determined based on the calculated data (Network_Congestion, Network_Error_Rate), which are obtained by passing through the previous Network congestion and Quality of Channel estimation procedures. Transmission policy and Ack policy are determined by the System manager from the Policy Table. Figure 9 shows an example of Policy Table. In this paper, an adaptive technique applicable to environments subject to severe changing of many parameters is introduced. In case of the Policy Table of Figure 9, threshold values classifying each level (MID or HIGH) are not determined. It is because those threshold values will be setup dynamically against to sea environment considering the reliability of data transmission and the system manager’s intentions. In other words, if perfect transmission is required, RBA method rather than no acknowledgement messages policy can be selected in case that congestion is MID and error rate is HIGH. Research on how to determine an optimal threshold value by using the data collected during network initiation is underway. In this paper, the failure rate and the anticipated data transmission capacity is obtained by comparing to the network capacity (bandwidth) and these will be changed also according to the performance of underwater communication instruments (20 kbps bandwidth is assumed in the simulation).

### 3.6. An Example of Smart Calculation Procedure

The procedure for determining various policies and output values is described below. When the propagation delay value of each node is input, the required value can be deduced by going through each process. Tables 1–3 show the variable definitions.

#### 3.6.1. Network Congestion Estimation Process

In Network Congestion estimation process, *Trans_rate* is calculated.

#_of_Slave = 10; // the number of Slaves

MPDU_Default_Length = 160; // Length of MAC frame (20 * 8 bits)

Trans_count_per_sec = 5; // # of frames per second by one Slave

Network_Capacity_bps = 20480; // (20 * 1024) bps

Trans_rate = (#_of_Slave) * (MPDU_Default_Length) * (Trans_count_per_sec) / (Network_Capacity_bps)

/* Trans_rate = 10*(20*8bit)*5/20*1024 = 8000/20480 = 0.39, the range is from T_HIGH_TH to T_MID_TH (refer Tab. 11)

Network_Congestion = MID */

IF (TV_HIGH_TH =< Trans_rate) THEN Network_Congestion=V_HIGH

ELSE_IF (T_HIGH_TH =< Trans_rate < TV_HIGH_TH)

THEN Network_Congestion=HIGH

ELSE_IF (T_MID_TH =< Trans_rate < T_HIGH_TH) THEN Network_Congestion=MID

ELSE_IF (T_LOW_TH =< Trans_rate < T_MID_TH) THEN Network_Congestion=LOW

#### 3.6.2. Quality of Network Estimation Process

In Quality of Network estimation process, Error_rate is calculated

BER = 0.3 // 30%

Self_error_Correction_Rate = 0.8 // FEC + CRC

Error_rate = BER * (1-Self_error_Correction_Rate)

/* Error_rate = 0.3* (1-0.8) = 0.06, Since the value is between E_MID_TH and E_LOW_THm, Network_Error_Rate is LOW. */

IF (EV_HIGH_TH <= Error_rate) THEN Network_Error_Rate=V_HIGH

ELSE_IF (E_HIGH_TH <= Error_rate < EV_HIGH_TH)

THEN Network_Error_Rate=HIGH

ELSE_IF (E_MID_TH <= Error_rate < E_HIGH_TH) THEN Network_Error_Rate=MID

ELSE_IF (E_LOW_TH < Error_rate <= E_MID_TH) THEN Network_Error_Rate=LOW

ELSE Network_Error_Rate=CLEAR

#### 3.6.3. Scale of Network Estimation Process

In Scale of Network estimation process, interval and time assignment values are calculated. DG List, Gain-time, which can be applicable to each link, and Guard-time are calculated.

/* For Network_Scale calculation, Max() function is conducted. Max() function returns the maximum value among Propagation_Delay[]. The value of 1000.0 ms is returned and divided by 2 to calculate one-way transmission time, not Round Trip time. Distance() function is called to determine Network_Scale. Distance() function measures the radius of overall Network by referring Network_Capacity_bps value, which is overall network capacity. In this example, the radius of Distance(1000/2, 20kbps), that is the maximum distance for transmission is about 750 m. It can be converted to 65 Time Slots.*/

Max(Propagation_Delay[ ])=1000.0

Network_Scale = Distance (Max (Propagation_Delay[ ]) / 2, Network_Capacity_bps)

/* Procedure of calculating Guard time until finishing Beacon transmission. Beacon message transmitting time to the most distant Slave is about 500ms(750m). It is 65 in Time Slot unit. */

Beacon_Guard_time = BGT(Network_Scale)

/* Equation for calculating Gain time in time slot unit. 150m is obtained by dividing 750m with 5 Distance Group. Taking acoustic wave propagation time of 1.5 km/s into account, it is 0.1 second (100ms) interval and unit time by transmission time difference between Distance Group is calculated at 12 (100ms / 7.5ms) */

GT_value = GT(Network_Scale)

/* If ±5% of transmission time variation is assumed, data transmission time to the most distance Slave can be 525ms (=500ms+(500ms*0.05)). At least 4 Time Slot (=25ms/7.8ms) should be secured for Guard time which should be assigned additionally. This value is reduced to 3, 2 and 1 as the distance interval is reduced from max. 4. depending on Distance Group. */

GTL_value = GTL(Network_Scale)

/* TDMA_interval can be determined taking the number of frame per second, network radius, guard time and the required slot for gain time into consideration and it is liquid. For example, the sum of Propagation delay(60), Guard time(4) and the required time for data transmission(1(normal data) ∼ 8(blocked data)) is 72 slot. In ideal condition of Gain Time, it can be reduced down to 48 slot. 72 slot is assumed if Gain time is nod considered. */

TDMA_interval = Interval (Network_Scale, GT_value, GTL_value, Trans_count_per_sec)

/* Beacon_interval can be a sum of TDMA_interval * #_of_Slave and Beacon_Guard_time at the finishing moment of Round for transmission. 72*20+60=204 slot */

Beacon_Interval = Interval (Network_Scale, TDMA_interval, #_of_Slave) + Beacon_Guard_time

#### 3.6.4. Smart Calculation Process

In Smart Calculation Process, the mode of ACK and Transmission using Policy-Table are determined and the NAV flag setting for reservation of transmission is determined.

/* If Network_Congestion is V_HIGH, it means there are very high network loads that it is impossible to secure the required transmission time. If Network_Error_Rate is V_HIGH, it means the error rate is too high so the traffic generated by transmission and re-transmission can not be executed in a stable manner. In case of setting up NAV, there are 8 NAV states according to the level of Error_Rate. The highest and lowest value is 0111 and 0000 respectively. In this example, the below procedure is not executed since Error_Rate is 0.39. */

IF (Network_Congestion==“V_HIGH” || Network_Error_Rate=“V_HIGH”)

 THEN NAV_state ()

NAV_state(){

 IF (Error_rate > 0.9) NAV_Flag=0111

 ELSE_IF (Error_rate > 0.8) NAV_Flag=0110

 .....

 ELSE NAV_Flag=0000

}

The values of Network_Error_Rate and Network_Congestion calculated from the Network Congestion estimation Process determines the policy of transmission and error restoration according to the policy determination table shown in Table 3. In this example, since Network_Congestion is MID and Network_Error_Rate is LOW, the policy is Blocked data Transmission with Multiple Blocked ACK transmission.

#### 3.6.5. Output Parameter

Results from the above process are listed in Tables 4 and 5.

## 4. Performance Analysis

### 4.1. Comparison

Presently, analysis and verification on various variables and parameters have been conducted based on a theoretical concept of the sea environment. For verification of the efficiency and reliable transmission policy of the proposed technique, the transmission collision probability of the UWSN-MAC technique which was proposed by Min Kyoung Park in 2007 was selected for comparison [9]. For the comparison, the Propagation delay was assumed as 0 to obtain packet collision probability excluding other parameters.

For the fraction of receive energy wasted due to collisions, the metric is a more accurate description of the receive energy usage, since the receive energy is indeed associated with only that receiver and no others. Hence, we measure the receive energy consumed by a receiver for the failed deliveries due to collisions. The fraction of receive energy wasted is at most equal to the fraction of transmit energy wasted (however, the magnitudes of the two energies can be dramatically different). Note that for the special case in which all the packets have the same duration, this fraction of receive energy wasted due to collisions reduces to the collision rate, which is the total number of packets that collide at receivers divided by the total number of packet transmissions attempted. Hence, tracking the fraction of receive energy wasted is more general and can be specialized if necessary.

In this section, we assume that all nodes share the same medium so that all of them can hear from each other. In the absence of propagation delays, we compute the probability that a collision occurs. Notations in equations are summarized in Table 6.

(1) $$\mathrm{Pc}=1-\mathrm{Pnc}=1-\left[{\left(1-\frac{2\tau }{T}\right)}^{N-1}\right]$$

(2) $$W=N(1-\left[{\left(1-\frac{2\tau }{T}\right)}^{N-1}\right])E$$

(3) $$\mathrm{Sum}(W)=N(1-\left[{\left(1-\frac{2\tau }{T}\right)}^{N-1}\right])E\cdot \mathrm{life}\_\mathrm{time}$$

Equation 1 is the probability of collision in UWSN-MAC technique and Equation 2 is the expected value of the total transmits energy wasted due to collisions. In the compared paper, the model is a computational model for collision probability with very small duty cycle regardless of network configuration. The model proposed in this paper, however, the *Ad hoc* technique can be considered in addition to a center-control technique with very low collision probability. Therefore, the authors wanted to consider network configuration load in mathematical form. In addition, in the initial stage of network configuration, there is no need to maintain data transmission duration but the network size, which is equivalent to required transmission time according to transmission distance, should be maintained. In this model, unit transmission distance is assumed and the effect of propagation delay is excluded. Besides, the collision probability during network configuration and the amount of energy waste were calculated as follows. *Pc’* and *Pnc’* indicate the collision probability and the non-collision probability respectively. *T_c_* is starting time of network configuration and *τ_c_* is transmission duration time for network configuration. This time will be determined to be proportional to transmission distance.

In this paper, time period of *T* and *T_c_* is represented as a rate of [0, *T*)/[0, *T_c_*) and [0, *T*)/[0, *T_c_*) ≤ 1. In case of [0, *T*)/[0, *T_c_*) = 1, as the mechanism of [6], it means that channel occupation and synchronization is conducted whenever communication is attempted. In case of [0, *T*)/[0, *T_c_*) < 1, it means one synchronization and many scheduling of communication period. This is the proposed technique. According to this rate, *config_time* which is proportional to *life_time* can be obtained. In the following equation, *int()* is a integer function:

(4) $$\mathrm{config}\_\mathrm{time}=\text{int}(\mathrm{life}\_\mathrm{time}/([0,T_{c})/[0,T)))$$

(5) $${\mathrm{Pc}}^{\prime }=1-{\mathrm{Pnc}}^{\prime }=1-\left[{\left(1-\frac{{2\tau }_{c}}{T_{c}}\right)}^{N-1}\right]$$

In the proposed technique, the collision probability is calculated as 0 because the each schedule for data transmission is already determined by the network configuration process. Of course, entry of new nodes and withdrawal of existing nodes can also be considered to construct a more reliable model in the future. The following formula is for calculation of energy consumption in the proposed technique. In the formula, *config_time* is smaller than *life_time* and W is the amount of energy consumption during unit transmission time:

(6) $$\mathrm{Sum}(W^{\prime })=N(1-\left[{\left(1-\frac{{2\tau }_{c}}{T_{c}}\right)}^{N-1}\right])E\cdot \mathrm{config}\_\mathrm{time}$$

**Figure 10.**
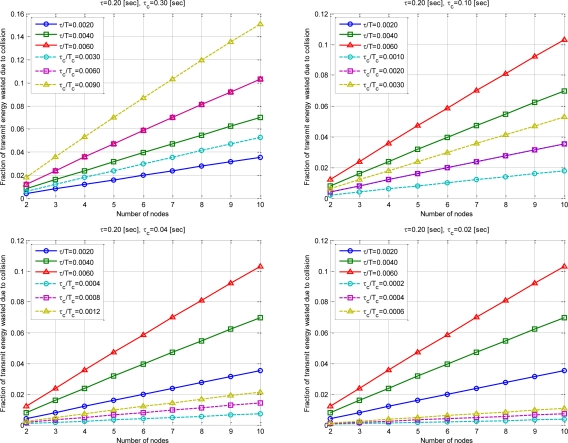
Fraction of transmit energy wasted due to collision.

The following are the comparison results of the conventional method [6] and the given mathematical model. Figure 10 shows the comparison results of 6 duty cycle *τ/T*, *τ_c_*/*T_c_* when a sensor node attempts to communicate. The energy waste by transmission packet collision is *τ_c_* value is 0.3, 0.1, 0.04, 0.02. It is the term of $(1-\left[{\left(1-\frac{2\tau }{T}\right)}^{N-1}\right])E$ of the formula.

**Figure 11.**
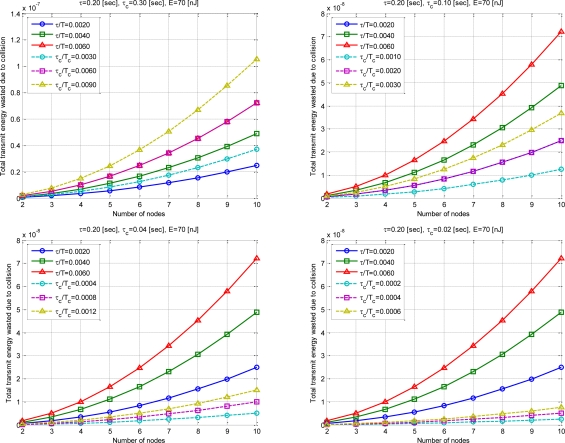
Total transmit energy wasted due to collision.

Figure 11 is the case of Total considering all sensor nodes. It means the value of $W=N(1-\left[{\left(1-\frac{2\tau }{T}\right)}^{N-1}\right])E$. One transmission energy consumption E is assumed as 70 nJ [9].

**Figure 12.**
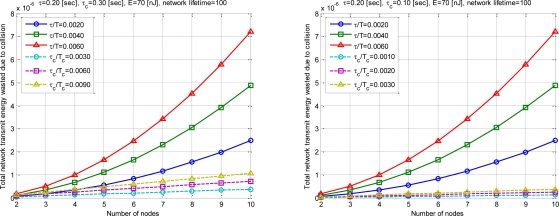

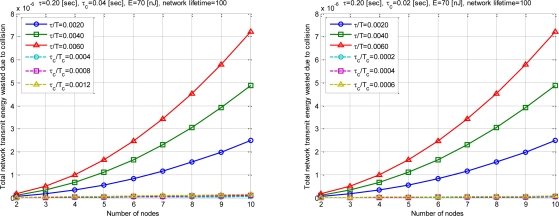
Total network transmit energy wasted due to collision.

Figure 12 is the summed energy waste value during network life time. Repetition of conduction number is multiplied. Formula’s conventional technique and newly proposed technique is meant by *Sum(W)* and *Sum(W’). Life_time* is assumed as 100.

**Figure 13.**
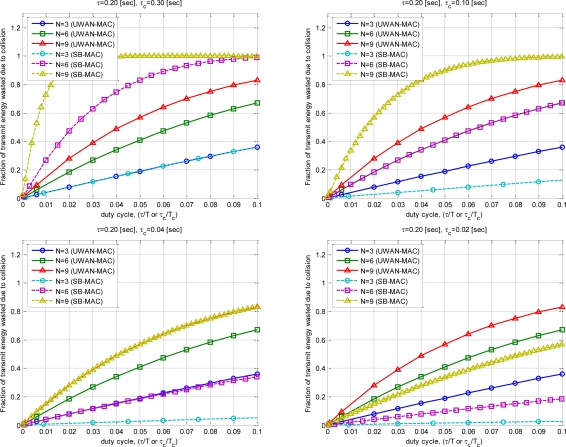
Energy wasted due to collision (# of node).

Figure 13 shows the variation according to the number of sensor node. The energy waste is compared according to duty cycle in case of the number of sensor node is 3, 6 and 9.

Simulation results showed that the energy efficiency can be significantly increased as *τ_c_* decreases with the proposed SBMAC. The case of *τ_c_* of 0.3 when *τ* is greater than *τ_c_*, network initialization is greater than transmission period. Therefore in the real world, it is not used. However, the case is inserted for simple numerical analysis results.

### 4.2. Simulation

For performance verification of the proposed method, an Omnet++-based Underwater MAC system was constructed. The underwater simulation environment characteristics are listed in Table 7. In Transmission mode, conventional methods of ARQ and Block Ack technique were compared with the proposed SB-MAC technique. ARQ transmits ACK one by one while Block ACK transmits many ACKs at a time. In this paper, ARQ, as the object of comparison, is a Stop and Wait-type ARQ. Since the sequence and number of transmission is determined by Master, we used simple ARQ rather than using more complicated ones, such as sliding window-type, Go-back-N or Selective repeat ARQ.

The simulation procedure includes two execution procedures, which are network configuration and data transmission procedure, proposed in the analytical model. However, it does not seem enough to explain the probability of analytical model results and simulation results which is focused on the application of various transmission modes and SB-MAC acknowledgement. As for the request of simulation results which are related to practical performance (throughput, success ratio), the following additional explanation and results are added. For analysis of the simulation results, the information of various factors was collected and summarized in Table 8. This information was collected through the whole simulation processes, printed at the ending stage of final simulations and used as data for analysis.

The following table shows the performance analysis of the proposed SB-MAC. In case of 0.1/sec, the sensor node’s delay time has the largest value in spite of low transmission interval, and it is because of significant transmission delay caused by application of the aggregation technique. In Table 9, the Success Ratio of MAC and PHY layer are listed, respectively. In this paper, Sink nodes play a role of notifying both transmission sequence and synchronization time of Sensor nodes by broadcasting Beacons periodically, and do not transmit data. Therefore, there are no accumulated packets in Queue. On the other hand, Sensor nodes transmit the aggregated data according to the determined time and order via Beacons received from Sink nodes. In this case, the aggregated data in transmission Queue increase as Transmission interval decrease. Therefore, the Queue Max value is exceeded after 3/sec and no performance is increased. In case of Throughput, the link of Sink and one of nine Sensor nodes was measured (in the simulation, there are one Sink and nine Sensors within a unit area).

**Figure 14.**
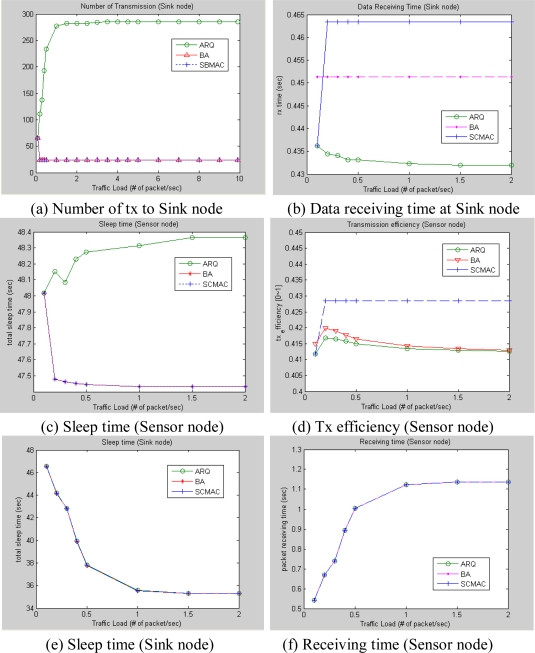
Simulation results.

Figure 14a shows the comparison results of number of transmission from the sink and sensor nodes’ point of view. Since cluster-based sea environment monitoring is assumed in this simulation, it can be observed that the number of transmission of sensor node is much smaller than that of the sink node. In the case of ARQ having a larger number of transmissions, duty cycle and energy consumption increased significantly and channel utilization was consequently decreased. Figure 14b shows the data transfer time. It is shown that SBMAC is best in its efficiency and other two techniques show the same result. This results show that the system operation time for Block Ack and data aggregation was increased. Figure 14d shows the efficiency of transmission by the transmitted data versus overall network usage. It was shown that SBMAC is the best technique and shows constant results regardless of Traffic load. Figure 14e,f has some interrelationships. In case of sink node, processing time is increased and Sleep time is decreased as Traffic load increased. However, the amount of transmitted control packets increases.

## 5. Conclusions

For underwater communication, various environment and variables should be considered. Recently, research to solve the problems of limited resources, high error rates and long transmission delay times has been undertaken. In this paper, a SBAC (Smart Blocking MAC) technique, which can significantly decrease the number of transmissions and change its transmission and retransmission policy adaptively against various environments, was proposed. Based on quantitative analysis results by mathematical modeling and performance analysis results by simulations, the propose technique showed the best performance compared with conventional techniques. The proposed method was more efficient, especially in the case where the network environment changes over a wide range or the number of nodes increases. Further research to increase its transmission efficiency using various configuration values according to Network Scale will be conducted.

## Figures and Tables

**Figure 2.**
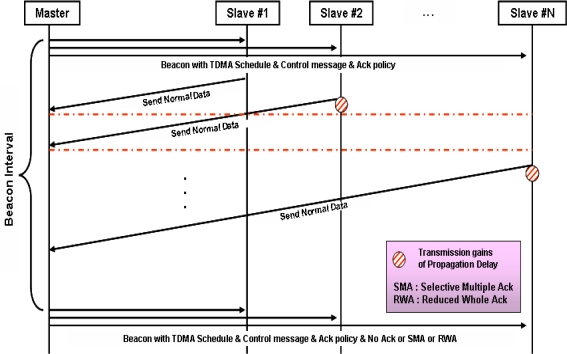
Normal data transmission.

**Table 1.** Sample of UW-ASN system input parameter.

**Parameter**	**Value**	**Parameter**	**Value**
# of Slave	10	TV_HIGH_TH	0.5
BER	0.3	T_HIGH_TH	0.4
Max. Propagation Delay	1 Sec	T_MID_TH	0.3
MPDU_default_Length	20 Byte	T_LOW_TH	0.2
Trans_count_per_sec	5	EV_HIGH_TH	0.2
Network_Capacity_bps	20 Kbps	E_HIGH_TH	0.15
Self_Error_Correction_Rate (FEC+CRC)	0.8	E_MID_TH	0.1
Time Slot	7.8 ms	E_LOW_TH	0.05

**Table 2.** Sample of Slave's Propagation_Delays.

**Slaves**	**Propagation_Delay (ms)**	**Slaves**	**Propagation_Delay (ms)**
Slave #01	1.5	Slave #06	520
Slave #02	24.5	Slave #07	603.8
Slave #03	110	Slave #08	617.9
Slave #04	300.2	Slave #09	800
Slave #05	376	Slave #10	1000

**Table 3.** Sample of Policy-Table.

**Network_Congestion**	**Network_Error_Rate**	**Transmission Policy**	**ACK Policy**
HIGH	HIGH	Blocked_Data	No_ACK
	MID	Blocked_Data	No_ACK
	LOW	Blocked_Data	No_ACK
	CLEAR	Blocked_Data	No_ACK
MID	HIGH	Blocked_Data	No_ACK
	MID	Blocked_Data	No_ACK
	LOW	Blocked_Data	Blocked_ACK
	CLEAR	Blocked_Data	Blocked_ACK
LOW	HIGH	Blocked_Data	No_ACK
	MID	Blocked_Data	Blocked_ACK
	LOW	Blocked_Data	Blocked_ACK
	CLEAR	Normal_Data	Normal_ACK
CLEAR	HIGH	Normal_Data	No_ACK
	MID	Normal_Data	Normal_ACK
	LOW	Normal_Data	Normal_ACK
	CLEAR	Normal_Data	Normal_ACK

**Table 4.** DG list.

**Slaves**	**Propagation_Delay (ms)**	**DG**	**Guard-Time (Time slot)**	**Gain-time (Time slot)**				
				**1**	**2**	**3**	**4**	**5**
Slave #01	1.5	1	0	0	−12	−24	−36	−48
Slave #02	24.5	1	0	0	−12	−24	−36	−48
Slave #03	110	1	0	0	−12	−24	−36	−48
Slave #04	300.2	2	1	12	0	−12	−24	−36
Slave #05	376	2	1	12	0	−12	−24	−36
Slave #06	520	3	2	24	12	0	−12	−24
Slave #07	603.8	4	3	36	24	12	0	−12
Slave #08	617.9	4	3	36	24	12	0	−12
Slave #09	800	4	3	36	24	12	0	−12
Slave #10	1000	5	4	48	36	24	12	0

**Table 5.** Sample of UW-ASN system output parameter.

**Parameter**	**Value**
Transmission Policy	Blocked Data Transmission
ACK Policy	Multiple Blocked ACK
Beacon Interval (Time Slot)	204
TDMA Interval (Time Slot,)	24 ∼ 72
Gain-time Info. (Time Slot)	0 ∼ 48
Guard-time Info. (Time Slot)	0 ∼ 4
NAV_Flag	1000

**Table 6.** Notation.

**Notation**	**Definition**
[0, *T*)	Each node generates its Transmission start time uniformly in the interval [0, *T*) in an independent identically distributed
*τ*	Data transmit duration (200 ms)
*Pc, Pc’*	The probability that a node’s transmission collides with at least another node’s transmission over 1 cycle
*Pnc, Pnc’*	The probability that a node’s transmission dose not collides over 1 cycle
*N*	Number of nodes in this network
*τ* / *T*	Duty cycle for data transmission
*τ_c_* / *T_c_*	Duty cycle for network configuration
*E*	Value of the energy for each transmission
*NE*	Total value of the transmit energy used for all nodes
*W*	Energy wasted due to collisions and Configuration
*Sum(W)*	Summing function of energy consumption by collisions during lifetime of the network
*life_time*	Lifetime of the network—the number of transmittable region
*config_time*	The number of reconfiguration region during lifetime of the network

**Table 7.** Simulation environment.

**Policy / Factor**	**Value**
Ack	ARQ, Block Ack, SBMAC
Number of Default Blocking	7
Network Topology	Infra - structured Network
Depth	500∼3,000 m
Temperature	0∼25 °C
Salinity	20∼40‰
Cluster Head	1∼4
Relay Node	Yes/No

**Parameter**	**Value**	**Parameter**	**Value**
# of Slave	10	TV_HIGH_TH	0.5
BER	0.3	T_HIGH_TH	0.4
Max. Propagation Delay	1 Sec	T_MID_TH	0.3
MSDU_default_Length	20 Byte	T_LOW_TH	0.2
Trans_count_per_sec	0.1∼10.0 (19 cases)	EV_HIGH_TH	0.2
Network_Capacity_bps	20 Kbps	E_HIGH_TH	0.15
Self_Error_Correction_Rate (FEC+CRC)	0.9	E_MID_TH	0.1
Time Slot	7.8 ms	E_LOW_TH	0.05

**Table 8.** Various factors.

**Factors**	
Number of Data Tx	Number of Blocked Data Tx
Number of Data Rx	Number of Blocked Data Rx
Number of Beacon Tx	Spend time for Data Tx
Number of Beacon Rx	Spend time for Data Rx
Number of ACK Tx	Spend time for Overhead Tx
Number of ACK Rx	Spend time for Overhead Rx
Number of Max Queue (Up to 100)	Radio sleep time
Transmission Delay time	Radio Tx time
Number of Tx_Drop	Radio Rx time

**Table 9.** Performance of SB-MAC.

**Transmission Interval(# of Tx)**		**Throughput (MAC-bps)**	**Total Delay time (Sec)**	**Success Ratio (PHY-%)**	**Tx Queue Len. (Max = 100)**
0.1/sec	Sink	128.1	0.791549	75.28 (data)	0
	Sensor	17.2	10.930507	48.65(beacon)	0
0.5/sec	Sink	656.3	3.2027	90.43(data)	0
	Sensor	71.9	0.8986	34.61(beacon)	0
1/sec	Sink	790.6	11.8534	91.18(data)	0
	Sensor	71.9	0.8986	34.61(beacon)	19
2/sec	Sink	806.3	18.0183	91.17(data)	0
	Sensor	71.9	0.8986	34.61(beacon)	76
3/sec	Sink	812.5	20.2541	91.19(data)	0
	Sensor	71.9	0.8986	34.61(beacon)	100
5/sec	Sink	815.6	22.1395	91.12(data)	0
	Sensor	71.9	0.8986	32.36(beacon)	100
10/sec	Sink	815.6	22.1395	91.12(data)	0
	Sensor	71.9	0.8986	32.36(beacon)	100
